# Complete mitochondrial genome of the copepod *Sinergasilus undulates* (Copepoda: Poecilostomatoida)

**DOI:** 10.1080/23802359.2020.1870890

**Published:** 2021-03-26

**Authors:** Cong-jie Hua, Ming-yan Su, Zhi-wei Sun, Yuan-hong Lu, Jin-mei Feng

**Affiliations:** a Wuhan Institute of Biomedical Sciences, School of Medicine, Jianghan University, Wuhan, People’s Republic of China; b Department of Pathogenic Biology, School of Medicine, Jianghan University, Wuhan, People’s Republic of China

**Keywords:** *Sinergasilus undulatus*, Copepoda, mitochondrial genome, phylogenetic analysis

## Abstract

The total mitochondrial genome size of *Sinergasilus undulatus* is 14,239 bp in length, including 13 protein-coding genes (PCGs), two rRNA genes, 22 transfer RNA genes, and a non-coding control region (D-loop). The overall nucleotide composition of the mitochondrial DNA of *S. undulatus* is 34.9% A, 35.5% T, 15.7% C, 13.9% G, and 70.4% AT, respectively. Phylogenetic analysis suggests that the genus *Sinergasilus* is monophyletic, and *S. undulatus* is closely related to *S. polycolpus*. The complete mitochondrial genome of *S. undulatus* would be useful for species identification, epidemiology, and phylogenetics among Copepods.

Copepods in the genus *Sinergasilus* Yin, 1949 are considered as the most important parasites in freshwater fish. In this genus, three species are described with restricted host specificity. Among them, *S. undulatus* is reported only infecting on common carp, *Cyprinus carpio*, and crucian carp, *Carassius auratus*, which are important economic fish species in China (Nie and Yao 2000). The first valid mitochondrial genome in the genus *Sinergasilus* Yin, 1949 is *Sinergasilus polycolpus* (Peng et al. 2010; Feng et al. 2015). Here, we assembled the complete mitochondrial genome of *S. undulatus*, which is the second reported one in this genus. The complete mitochondrial genome of *S. undulatus* would be useful for species identification, epidemiology, and phylogenetics among Copepods.

In this study, the *S. undulatus* was collected from a naturally infected *C. carpio*, obtained in the Lake Dongting, Yueyang (29°38′N, 113°09′E), China. The complete mitochondrial gene sequences were determined using the Sanger sequencing method. Voucher specimens are permanently stored in absolute ethanol under accession number WIBS20150911, in Wuhan Institute of Biomedical Sciences, School of Medicine, Jianghan University, Wuhan, China. The total genomic DNA was extracted from a single specimen (WIBS 20150911_1) using SDS/Proteinase K according to the instructions of TIANamp Genomic DNA kit (Tiangen Biotech, Beijing, China) following the manufacturer’s protocol. Subsequently, based on the existing mitochondrial genes of *S. polycolpus* (EU621723, NC_028085), pairs of primers were designed to amplify fragments from *16S rRNA* (F: 5′-CTTAATTCAACATCGAGGTC-3′, R: 5′-TAGACGAGAAGACCCTA-3′), *cox1* (F: 5′-GTAAHCACAADGATATTGGTAC-3′, R: 5′-CGHCGNGGTATGCCTGCTAACC-3′), *cox2* (F: 5′-CAAATTCTAAAGCGATAGG-3′, R: 5′-GATTTTGTTATAACAATCTTGG-3′), and *cytb* (F: 5′-GTTATGTTCTACCTTGDGGNCA-3′, R: 5′-TCTACTGGNCGTGCHCCGATTC-3′). Then, other pairs of primers were designed to amplify the remaining mitochondrial genome sequences of *S. polycolpus*, *16S-cox3* (F: 5′-GAAATTGATTAGTGCTCATTTTC-3′, R: 5′-CTACTGGGTTTCATGGATTAC-3′), *cox3* (F: 5′-CACGCTGCVGCNTCAAAACC-3′, R: 5′-GTAGATGAGTCTCCATGACC-3′), *cox3-cox1* (F: 5′-CATCTCGTCATCATTGATAC-3′, R: 5′-GCATGGGCGGTTACGATTAC-3′), *cox1-cox2* (F: 5′-GCTAGTCCACTGGTACCCAC-3′, R: 5′-GATTAGGGGTTAAGGCTGAC-3′), *cox2-nad5* (F: 5′-CTAAAGCAGGGATTAAAGTTC-3′, R: 5′-GTAATAACCACATCTCTATAG-3′), *nad5* (F: 5′-CCTGCTGCAATAGCNGCNCC-3′, R: 5′-CCTAANTGTCTHAAAGTTGA-3′), *nad5-cytb* (F: 5′-ATGAACTGGAAGGATAAC-3′, R: 5′-GTTAAGGTAGCGTTGTTTACTG-3′), and *cytb-16S rRNA* (F: 5′-CAAAACTAGTTTAACAAAGAG-3′, R: 5′-GGTACTTTAGGGATAACAGC-3′). The samples were amplified by PCR, and then sequenced using Sanger sequencing technology. The complete mitochondrial sequences were assembled manually and aligned against other published mitochondrial genome sequences of Copepods using the program MAFFT 7.149 (Katoh and Standley 2013) to determine the gene boundaries. BLAST and ORF Finder NCBI tools were also used to identify and annotate the protein-coding genes (PCGs) and rRNAs. Transfer RNA (tRNA) genes and their secondary structures were identified using tRNAscan-SE 1.21 (Lowe and Eddy 1997), MITOS (Bernt et al. 2013), and ARWEN 1.2 (Laslett and Canback 2008). Nucleotide composition (%) of the complete mitochondrial sequences was calculated using PhyloSuite v1.2.2 (Zhang et al. 2020). The phylogenetic analysis and analysis of other nucleotide and amino acid components were performed by using PhyloSuite v1.2.2 (Zhang et al. 2020). The complete mitochondrial genome sequence of *S. undulatus* (GenBank accession number MW080644) is 14,239 bp in length, including 13 PCGs, two rRNA genes, 22 tRNA genes, and a non-coding control region (D-loop). The overall nucleotide composition of its mitochondrial DNA is 34.9% A, 35.5% T, 15.7% C, 13.9% G, and 70.4% AT, respectively. Seven PCGs started with ATA, three PCGs started with ATG, but *cytb* uses ATC, *nad3* uses ATT as the start codon; nine PCGs were finished with TAA, but nad4 and nad6 were finished with TAG. The incomplete stop codon (T––) was found in two genes (cox2 and cox3). Fourteen overlaps exist among mitochondrial genes. All 22 tRNAs distributed on the H and L strands were between 53 and 74 bp in length. Fifteen tRNA genes were encoded on the L and seven on the H strands. Most of the tRNAs could form a common cloverleaf secondary structure, with the exception of three tRNAs that lacked the DHU arm (trnR, trnS_1_, and trnS_2_), and the trnC lacking the TψC arm. Two rRNA genes, *12S* and *16S* were 657 bp and 919 bp in size, respectively.

A phylogenetic tree was reconstructed using 18 mitogenomes from the subclass Copepoda and two species of Branchiopoda. In PhyloSuite, amino acid sequences and two rRNA genes were aligned in batches and ambiguously aligned fragments removed using two plug-in programs: MAFFT (Katoh and Standley 2013) and Gblocks 0.91b (Talavera and Castresana 2007), respectively. Subsequently, the optimized alignments were concatenated by PhyloSuite. Bayesian information criterion in ModelFinder (Kalyaanamoorthy et al. 2017) was used to select the optimal evolutionary model (GTR + F+I + G4). Bayesian inference analysis, conducted using MrBayes 3.2 (Ronquist et al. 2012), was used for phylogenetic reconstruction: 2,000,000 generations, four Markov chain Monte Carlo chains, and the trees were sampled every 1000 generations, and the initial 25% of trees were discarded as burn-in. The phylogenetic tree suggested that the genus *Sinergasilus* is monophyletic, and the *S. undulatus* is the sister group to *S. polycolpus* (Figure 1).

**Figure 1.**
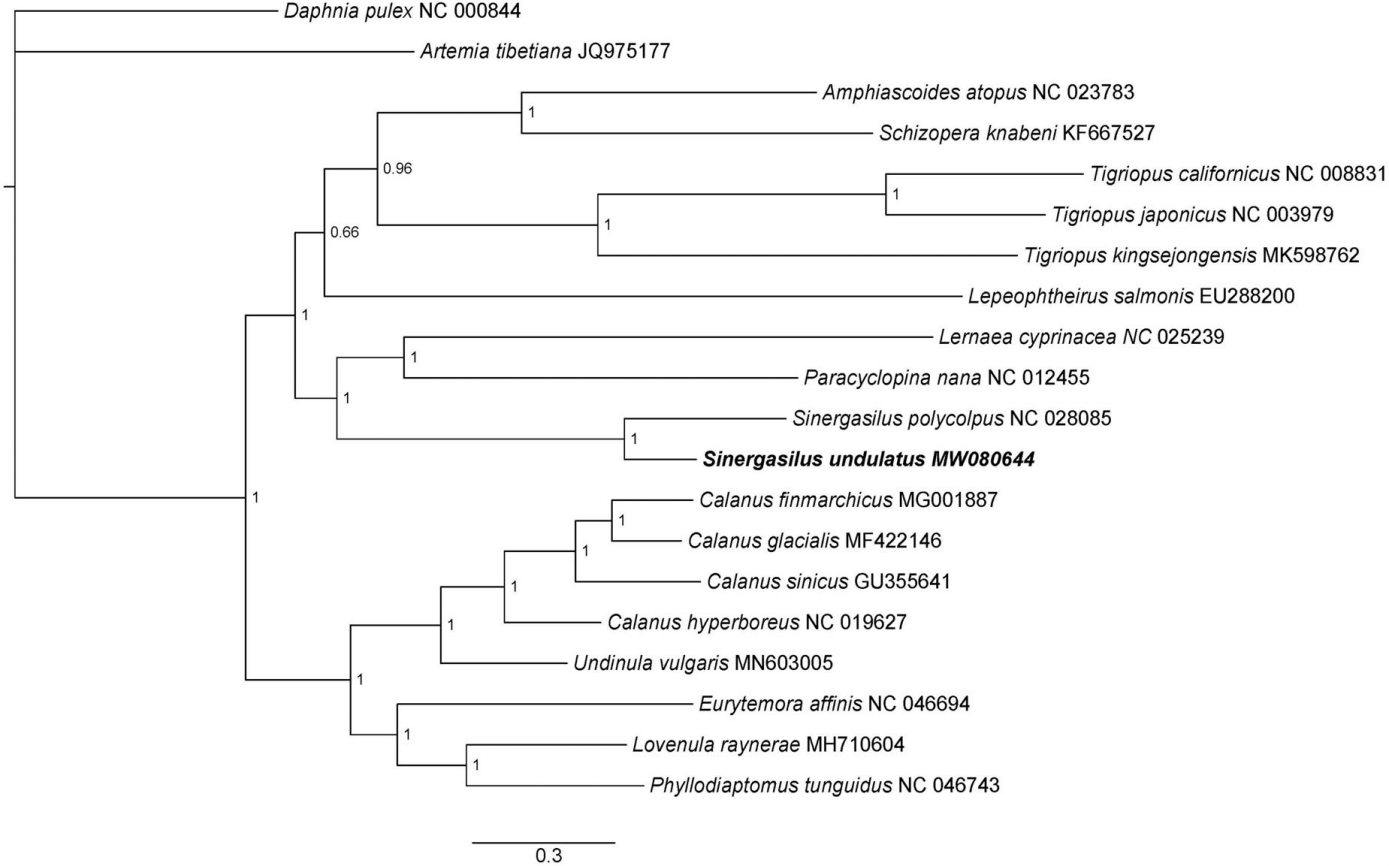
Phylogenetic tree for copepod mitochondrial genomes and two outgroup species from Branchiopoda was inferred by using Bayesian’s inference analyses based on concatenated nucleotide sequences of 13 mitochondrial protein-coding genes and two rRNA genes. Numbers next to nodes indicate Bayesian posterior. The scale bar indicates evolutionary distance.

## Data Availability

Mitogenome data supporting this study are openly available in GenBank at: https://www.ncbi.nlm.nih.gov/nuccore/MW080644.
